# Food Policy Councils and Healthy Food Access Policies: A 2021 National Survey of Community Policy Supports

**DOI:** 10.5888/pcd22.240335

**Published:** 2025-01-09

**Authors:** Reena Oza-Frank, Amy Lowry Warnock, Larissa Calancie, Karen Bassarab, Anne Palmer, Kristen Cooksey Stowers, Diane Harris

**Affiliations:** 1 Division of Nutrition, Physical Activity, and Obesity, National Center for Chronic Disease Prevention and Health Promotion, Centers for Disease Control and Prevention, Atlanta, Georgia; 2 Friedman School of Nutrition Science and Policy, Tufts University, Boston, Massachusetts; 3 Center for a Livable Future, Johns Hopkins Bloomberg School of Public Health, Baltimore, Maryland; 4 Department of Allied Health Sciences, University of Connecticut, Storrs, Connecticut

## Abstract

**Introduction:**

Food policy councils (FPCs) are frequently used to facilitate change in food systems at the local, state, and regional levels, or in tribal nations. The objective of this study was to describe the prevalence of food policy councils and similar coalitions among US municipalities and their associations with healthy food access policies.

**Methods:**

We used data from the 2021 National Survey of Community-Based Policy and Environmental Supports for Healthy Eating and Active Living, administered to municipal officials from May through September 2021. We used logistic regression models to examine associations between 1) having an FPC and 2) FPC membership composition and healthy food access policies. We grouped policies into 4 categories based on topic modules in the survey instrument: supporting new or existing food stores to sell healthy foods, financial or electronic benefits transfer (EBT) supports, transportation-related supports for accessing locations to purchase food, and consideration of local food supports in community planning.

**Results:**

Municipalities with FPCs (27.6%) had significantly higher odds than municipalities without FPCs of having policies supporting access to food retail stores (adjusted odds ratio [AOR] = 1.5; 95% CI, 1.2–1.9), access to farmers markets (AOR = 2.2; 95% CI, 1.7–2.7), access to transportation supports (AOR = 2.2; 95% CI, 1.8–2.8), and objectives in community planning documents (AOR = 2.0; 95% CI, 1.6–2.5). Among municipalities with FPCs, those with a health/public health representative (42.1%) or a community representative (65.1%) were more likely to report having any healthy food access policies.

**Conclusion:**

This study emphasized the positive association between FPCs and healthy food access policies. This study also highlights the potential importance of FPC membership composition, including health/public health and community representatives.

**Summary**

**What is already known on this topic?**

Municipalities with food policy councils (FPCs) and similar coalitions are more likely than municipalities without such entities to report having local supports for healthy food access.

**What is added by this report?**

This study shows the positive association between the presence of an FPC and the existence of health-promoting policies. It also highlights the importance of FPC membership composition, including health/public health and community representatives.

**What are the implications for public health practice?**

As FPCs work to maximize health benefits for populations that experience health and economic disparities, health/public health and community representatives can provide expertise to help FPCs evaluate the effect of their work.

## Introduction

The role of diet is important in chronic disease and obesity prevention (1,2). Widespread changes in individual dietary and lifestyle patterns could produce substantial gains in the population’s health (3), and are much more likely to occur in a supportive environment with accessible and affordable healthy food choices (3). Policy, systems, and environmental (PSE) change strategies can be used to create healthy food environments and achieve goals related to reducing chronic disease and preventing obesity (4,5).

Food policy councils and similar coalitions (hereinafter, collectively referred to as FPCs) are frequently used to facilitate change in food systems at the local, state, and regional levels, or in tribal nations (6,7). FPCs bring together representatives from various sectors (ie, agriculture, nutrition, business, government, and community) to work across the food system to express their values, initiate collective action, coordinate programs, and identify, establish, adopt, and implement food policy priorities (8–15). Since 2013, the Johns Hopkins Center for a Livable Future’s Food Policy Networks (FPN) project has “attempted to reach every FPC through a survey, conducted every 12-18 months, which asks about their status, structure, priorities, membership, challenges, and successes” (16). As of 2020 in the US, 283 FPCs were active in all but 3 states, with 68% operating at the local level (county, city/municipality, or both) (10).

While the missions of FPCs vary, most councils aim to increase access to healthy foods; address food and nutrition security; promote sustainable, local agriculture and economic growth; and/or encourage equity in food systems (8,9,14). These objectives are pursued through policies and programs to address issues such as poverty, economic development, food production, hunger, and public health (7). As such, FPCs may generate support from federal and state agencies and are recognized by the geographic area they serve as important collaborations that may help to improve local, state, and regional food systems and create healthier food environments (17).

Previous studies have measured the effectiveness of FPCs based on the degree of systems-level thinking and leadership toward influencing policy (8,14,15,18); advocacy (19); diversity, equity, and inclusion (11); or member empowerment, council credibility, synergy, and effect (measured as members’ perceptions of accomplishments) (14). However, studies assessing the effect of FPCs on PSE change have not directly measured policy adoption as an outcome of FPC efforts (6,11,14,15,19,20). Research based on 2014 data (12) found that municipalities with an FPC were more likely than municipalities without one to report having policy supports in place for healthy food access. The objective of our study was to update this previous study by using 2021 data from the National Survey of Community-Based Policy and Environmental Supports for Healthy Eating and Active Living (CBS-HEAL) to examine associations between 1) having an FPC and 2) FPC membership composition and healthy food access policies.

## Methods

The 2021 CBS-HEAL survey is a nationally representative, cross-sectional survey of US municipalities with populations of 1,000 or more and is conducted by the Centers for Disease Control and Prevention (CDC). Details on methods for the 2021 survey are available elsewhere (21). The CBS-HEAL survey was determined not to be human subjects research by CDC and thus did not need institutional review board approval.

Survey data were collected from May through September 2021, and sampled municipalities were drawn from the 2017 US Census of Governments (22). The survey was sent to the city or town manager, city planner, city administrator, or someone with similar responsibilities in each municipality. Respondents completed the survey with assistance from other municipal officials, if needed, via a secure website or paper version. The sampling frame included 10,300 municipalities. In 2021, the survey sampled 4,417 municipalities by using stratified random sampling by region and urban status; 1,982 municipalities completed the survey, corresponding to a 45% response rate.

To assess the presence of FPCs, respondents were asked, “Does your community have a local, county, or regional food policy council, food security coalition, or other community group working to increase access to healthy food?” (Table 1). A response of yes to this question indicated the presence of an FPC or similar coalition; responses of no and “don’t know” were considered not to have an FPC (12). If the respondent answered yes to having an FPC, follow-up questions on the membership composition were, “Is a local government employee or elected official a member of the food policy council, food security coalition, or other community groups working to increase access to healthy food?” and “Is there a designated health/public health or community representative on the food policy council, food security coalition, or other community groups working to increase access to healthy food?” (Table 1); response options were yes, no, or “don’t know” separately for a health/public health or a community representative. We excluded responses of “don’t know” from analyses.

**Table 1:** Survey Questions about Food Policy Councils (FPCs) or Similar Coalitions and Municipal Policies that Support Access to Healthy Foods, National Survey of Community-Based Policy and Environmental Supports for Healthy Eating and Active Living, United States, 2021

Topic area	Question
Food policy council	Does your community have a local, county, or regional food policy council, food security coalition, or other community group working to increase access to healthy food? (Objectives 1, 2, 3) If yes: a) Is a local government employee or elected official a member of the food policy council, food security coalition, or other community group working to increase access to healthy food? (Objective 3) b) Is there a designated health/public health or community representative on the food policy council, food security coalition, or other community group working to increase access to healthy food? (Objective 3) [Responses were recorded separately for a health/public health or a community representative.]

New supermarkets or existing convenience or corner stores	Does your local government currently use any of the following approaches to encourage supermarkets and other full-service grocery stores to open stores? a) Tax incentives (for example, tax abatement, tax credit, or property tax exemption) b) Grant or loan programs c) Programs to link store openings to broader neighborhood revitalization projects (for example, improvements to lighting, signage, safety, or walkability in the surrounding commercial corridor)
	Does your local government provide any of the following to help convenience or corner stores sell healthier foods? a) Grant or low-interest loan programs to purchase equipment for storage or sales of healthful foods (eg, refrigeration or a point of sale system) b) Technical assistance or training programs to increase the ability to sell healthier foods (eg, support for new point of sale systems, marketing assistance, produce handling training, product placement) c) Programs to link convenience or corner store improvements to broader neighborhood revitalization projects (improvements to lighting, signage, safety, walkability)

Farmers markets, farm stands, and green/produce carts	Does your local government have any policies related to farmers markets, farm stands, or green/produce carts that… a) Allow vendors to sell fresh produce on city-owned property b) Streamline processes for obtaining health and food safety permits and licenses c) Extend waivers of required business permits or retail licensing fees or taxes d) Provide funds or in-kind services for personnel, signage, or advertising e) Encourage opening in lower-income neighborhoods lacking supermarkets or full-service grocery stores
	Does your community have a farmers market, farm stand, or green/produce cart? If response is yes or no, but had one or more in the past, then asked: Does your local government provide funding for electronic benefits transfer (EBT) machines, or provide technical assistance on how to obtain or use EBT machines at local farmers markets, farm stands, or green/produce carts?

Transportation-related supports	When planning public transit, does your local government consider locating near the following destinations? a) Farmers markets b) Supermarkets or other full-service grocery stores
	Even if your community is not served by mass transit, does your local government operate paratransit community vans or shuttle buses that operate on as-needed or on-demand basis? If yes response, then asked: Do these vans or shuttle buses provide transportation to any of the following destinations? a) Farmers markets b) Supermarkets or other full-service grocery stores

Community planning documents	Does your local government have any of the following objectives included in the (comprehensive/general/master) plan(s)? a) Supporting farmers markets or community gardens b) Preserving land for agricultural uses

Respondents were also asked whether their communities had policies to support increased access to healthy foods (Table 1). These policies were grouped into 4 categories based on topic modules in the survey: 1) approaches to open new supermarkets or help existing convenience or corner stores sell healthy foods (categorized as food stores); 2) financial or electronic benefits transfer (EBT) supports for farmers markets, farm stands, and green/produce carts (categorized as farmers markets); 3) transportation-related supports for accessing supermarkets, other full-service grocery stores, or farmers markets (categorized as transportation-related); and 4) consideration of farmers markets/community gardens and agricultural land in community planning documents (categorized as community planning). For each of the 4 categories, the outcome variable of having any policy to support healthy food access was defined by a response of yes to at least 1 question in the respective category. The reference group comprised municipalities that responded no or “don’t know” (12).

Control variables used in this analysis were based on previous literature (12) and included municipal-level characteristics: population size, urban or not urban status, geographic region, median education level, poverty prevalence, and racial and ethnic composition. Municipal population size was categorized into 3 levels: 1,000 to 2,499; 2,500 to 49,999; and 50,000 or more. Municipalities were considered urban if 50% or more of the population resided in areas defined as urban based on the proportion of the population that resides within a census-designated urban area. Municipalities were also classified into the 4 geographic census regions: West, Northeast, South, and Midwest (23). The 2020 American Community Survey (24) was used to define the median education level of the population aged 25 years or older (≤high school diploma vs ≥some college) and the percentage of the population living below the federal poverty line (<20% vs ≥20%), to reflect persistent poverty as defined by the US Department of Agriculture (25), and the racial and ethnic composition of the population (≤50 vs >50% non-Hispanic White).

The final analytic sample size was 1,968 municipalities. We excluded from analysis 14 (0.7%) municipalities that completed the survey but were missing responses to the FPC question. We calculated the prevalence and associated 95% CIs of having an FPC overall and by municipal characteristics. Among municipalities with FPCs, we calculated the proportion that reported having representation from 1) a government employee/elected official, 2) a health/public health representative, or 3) a community representative, and 4) any of these officials with any policies to support increased access to healthy foods.

We used χ^2^ tests to assess differences in the prevalence of having an FPC by municipality characteristics. A *P* value of <.05 defined significance. We used logistic regression models to obtain odds ratios (ORs) of having an FPC and adjusted for municipality characteristics. We also used logistic regression to examine associations between having an FPC and the 4 categories of policies (food stores, farmers markets, transportation-related, and community planning) and their subquestions, adjusted for municipality characteristics. Finally, we used χ^2^ tests and logistic regression to assess associations between FPC membership composition and having any type of policy support for healthy food access.

Because 22% of municipalities responded “don’t know” to the question on having an FPC, we conducted a sensitivity analysis to test whether results differed when the reference group included responses of no only vs no or “don’t know” (ie, the primary analysis). Analyses were weighted to account for the complex survey design, including unequal probabilities of selection and varying nonresponse rates, using region and urban or not urban status to define weighting classes. We used survey procedures in SAS version 9.4 (SAS Institute Inc) to conduct all analyses.

## Results

Most municipalities had a population of 2,500 to 49,999 people (weighted frequency, 58.6%), were urban (75.5%), had a median education level of some college or more (67.7%), had a population in which less than 20% lived in poverty (78.7%), and had a majority non-Hispanic White population (83.2%).

Among US municipalities with at least 1,000 residents in 2021, 27.6% reported having a local or regional FPC (Table 2). The prevalence of having an FPC varied by municipal characteristics. Having an FPC was more common in municipalities with 50,000 or more people (54.3%) compared with those with 2,500 to 49,999 (28.7%) or fewer than 2,500 people (19.9%) (Table 2). After multivariable adjustment, municipalities with 50,000 or more people had 3.7 (95% CI, 2.3–5.9) times higher odds of having an FPC compared with municipalities with 1,000 to less than 2,500 people. FPCs were also more common in municipalities in which less than 50% of the population was non-Hispanic White than in those with a majority non-Hispanic White population (38.5% vs 25.4%), which remained significant after multivariable adjustment (adjusted OR [AOR] = 1.6; 95% CI, 1.2–2.2). Similarly, FPCs were more common in municipalities with a poverty prevalence of 20% or more (31.8%; AOR = 1.5, 95% CI, 1.1–2.0) than in municipalities with a poverty prevalence of less than 20% and in municipalities with a median educational level of some college or more (30.1%; AOR = 1.7; 95% CI, 1.3–2.2) than in municipalities with a median educational level of a high school diploma or less. FPCs were also more common in urban municipalities than in not urban municipalities (30.1% vs 20.3%); however, these differences were not significant after adjustment for other municipality characteristics. The distribution of municipalities with FPCs by geographic region was similar (Table 2).

**Table 2:** Prevalence and Adjusted Odds of Having a Local or Regional Food Policy Council (FPC) or Similar Coalition Among US Municipalities, by Municipality Characteristics, 2021^a^

Characteristic	No.	Yes, % (95% CI)	*P* value^b^	AOR (95% CI)^c^
**All municipalities^d^**	1,968	27.6 (25.6–29.6)	—	—
**Population size**				
1,000 to <2,5000	670	19.9 (16.8–23.1)	<.001	1 [Reference]
2,500–49,999	1,136	28.7 (25.9–31.4)		1.5 (1.0–2.1)
≥50,000	162	54.3 (46.6–62.1)		3.7 (2.3–5.9)^e^
**Urban/not urban status**				
Not urban	514	20.3 (16.6–24.0)	<.001	1 [Reference]
Urban	1,446	30.1 (27.6–32.5)		1.0 (0.7–1.4)
**Geographic region**				
Midwest	660	25.0 (21.7–28.3)	.13	1 [Reference]
Northeast	298	27.8 (22.4–33.2)		1.1 (0.8–1.5)
South	560	28.1 (24.4–32.0)		1.0 (0.8–1.3)
West	450	32.2 (27.8–36.7)		1.0 (0.7–1.3)
**Median educational attainment**				
High school diploma or less	596	22.3 (18.9–25.7)	<.001	1 [Reference]
Some college or more	1,372	30.1 (27.6–32.6)		1.7 (1.3–2.2)^e^
**Poverty prevalence, % of population below federal poverty level**				
<20	1,570	26.4 (24.2–28.7)	.04	1 [Reference]
≥20	398	31.8 (27.1–36.5)		1.5 (1.1–2.0)^e^
**Race and ethnicity, % of population non-Hispanic White**				
>50	1,648	25.4 (23.3–27.6)	<.001	1 [Reference]
≤50	320	38.5 (33.0–44.0)		1.6 (1.2–2.2)^e^

Abbreviations: — , does not apply; AOR, adjusted odds ratio.

a Data source: 2021 National Survey of Community-Based Policy and Environmental Supports for Healthy Eating and Active Living (21).

b Calculated by using χ^2^ test for differences in prevalence of having an FPC.

c Adjusted for municipal population size, urban/not urban status, geographic region, median education level, poverty prevalence, and race and ethnicity. Referent group was municipalities who reported no or “don’t know” to the question on having a food policy council.

d Excludes 14 municipalities with missing responses to the survey question, “Does your jurisdiction have a local or regional food policy council, food security coalition, or similar entity?”

e Significant based on the 95% CI not including the null value of 1.

Having an FPC was significantly associated with municipal policies to support improving access to healthy foods. Among municipalities with an FPC (n = 548), most (92.3%) reported having at least 1 policy for healthy food access, compared with 80.6% of municipalities without an FPC (Table 3). After multivariable adjustment, municipalities with an FPC had significantly higher odds than those without an FPC of having any policies (AOR = 2.5; 95% CI, 1.7–3.7), policies for food stores (AOR = 1.5; 95% CI, 1.2–1.9), supports for farmers markets (AOR = 2.2; 95% CI, 1.7–2.7), transportation-related policies (AOR = 2.2; 95% CI, 1.8–2.8), and objectives in community planning documents (AOR = 2.0; 95% CI, 1.6–2.5) (Table 3). Each policy in each category was significantly associated with the presence of an FPC.

**Table 3:** Prevalence and Adjusted Odds Ratios of Having a Policy to Improve Access to Healthy Foods Among US Municipalities^a^ (N = 1,968), by the Presence of a Food Policy Council (FPC) or Similar Coalition, 2021^b^

Type of support	Prevalence of policy supports			Has an FPC vs no FPC, AOR (95% CI)^d^
	Overall, %	Municipalities with an FPC (n = 548), % (95% CI)^c^	Municipalities without an FPC (n = 986), % (95% CI)^c^	
**At least 1 policy for healthy food access**	83.9	92.3 (90.2–94.9)	80.6 (78.5–82.8)	2.5 (1.7–3.7)
**New supermarkets or existing convenience or corner stores**	30.7	38.5 (34.2–42.7)	27.7 (25.4–30.1)	1.5 (1.2–1.9)
Supermarkets supports	28.6	35.0 (30.9–39.1)	26.1 (23.8–28.5)	1.4 (1.1–1.8)
Convenience or corner stores supports	8.6	17.3 (14.0–20.5)	5.3 (4.2–6.5)	3.2 (2.3–4.5)
**Farmers markets, farm stands, and green/produce carts**	61.3	74.8 (71.0–78.5)	56.2 (53.5–58.9)	2.2 (1.7–2.7)
Permitting or financial supports for farmers markets	60.5	73.5 (69.6–77.3)	55.6 (52.9–58.2)	2.1 (1.6–2.6)
Support for EBT at farmers markets, farm stands, or green/produce carts	8.3	16.7 (13.5–19.9)	5.1 (3.9–6.3)	3.0 (2.1–4.2)
**Transportation**	42.4	60.0 (55.7–64.2)	35.6 (33.2–38.1)	2.2 (1.8–2.8)
Consideration of locating public transport near farmers markets or supermarkets/other full-service grocery stores	28.7	44.6 (40.3–48.8)	22.7 (20.5–24.8)	2.2 (1.7–2.8)
Operate community vans/shuttle buses on as-needed or on-demand basis to farmers markets or supermarkets/other full-service grocery stores	30.4	42.4 (38.1–46.6)	25.4 (23.1–27.6)	1.7 (1.4–2.2)
**Community planning documents**	55.5	69.9 (65.9–73.9)	50.1 (47.4–52.8)	2.0 (1.6–2.5)
Farmers markets/community gardens	49	64.7 (60.5–68.8)	43.0 (40.3–45.6)	2.2 (1.7–2.7)
Preserving land for agricultural uses	25.1	32.0 (28.0–36.0)	22.5 (20.3–24.7)	1.4 (1.2–1.9)

Abbreviations: AOR, adjusted odds ratio, EBT, electronic bank transfer.

a Excludes 14 municipalities with missing responses to the survey question, “Does your jurisdiction have a local or regional food policy council, food security coalition, or similar entity?”

b Data source: 2021 National Survey of Community-Based Policy and Environmental Supports for Healthy Eating and Active Living (21).

c Differences between municipalities that reported having an FPC and those that reported no or “don’t know” were assessed by using χ^2^ test; all differences were significant at *P* < .05.

d Adjusted for municipal population size, urban/not urban status, geographic region, median education level, poverty prevalence, and race and ethnicity. Referent group was municipalities that reported no or “don’t know” to the question on having a food policy council. All AORs were significant based on the 95% CI not including the null value of 1.

Among municipalities with available data for both FPC and member composition, 44.0% (weighted %; 203 of 464) reported that they had a local government employee or elected official, 42.1% (weighted %; 179 of 392) reported that they had a designated health/public health representative, and 65.1% (weighted %; 282 of 425) reported having a community representative as a member of the FPC (Table 4). Municipalities who reported having a health/public health representative were significantly more likely to report having any policies to improve access to healthy foods (AOR = 4.7; 95% CI, 1.3–17.6), as were municipalities with a community representative (AOR = 4.5; 95% CI, 1.6–12.3) (Table 4). Sensitivity analyses that compared responses of yes and no yielded AORs that were similar in magnitude and significance to the AORs that compared yes and no or “don’t know.”

**Table 4:** Relationship Between Membership Composition of Food Policy Councils and Having Any Type of Policy Support for Healthy Food Access Among Municipalities That Reported Having a Food Policy Council or Similar Coalition, US, 2021^a^

Membership	Prevalence, % (95% CI)	AOR^b^ (95% CI)
Has a local government employee or elected official^c^		
Yes	44.0 (39.3–48.6)	2.3 (0.9–5.9)
No	—	1 [Reference]
Has a designated health/public health representative^d^		
Yes	42.1 (37.3–46.9)	4.7 (1.3–17.6)^e^
No	—	1 [Reference]
Has a community representative^f^		
Yes	65.1 (60.4–69.8)	4.5 (1.6–12.3)^e^
No	—	1 [Reference]

Abbreviations: — , does not apply; AOR, adjusted odds ratio.

a Data source: 2021 National Survey of Community-Based Policy and Environmental Supports for Healthy Eating and Active Living (21).

b Adjusted for municipal population size, urban/not urban status, geographic region, median education level, poverty prevalence, and race and ethnicity. Reference group was municipalities that reported no to the question on a council representative: “Is a local government employee or elected official a member of the food policy council, food security coalition, or other community groups working to increase access to healthy food?” or “Is there a designated health/public health or community representative on the food policy council, food security coalition, or other community groups working to increase access to healthy food?”

c Includes 464 municipalities with a yes response to the survey question, “Does your jurisdiction have a local or regional food policy council, food security coalition, or similar entity?” Excludes missing and “don’t know” responses to the survey question, “Is a local government employee or elected official a member of the food policy council, food security coalition, or other community groups working to increase access to healthy food?”

d Includes 392 municipalities with a yes response to the survey question, “Does your jurisdiction have a local or regional food policy council, food security coalition, or similar entity?” Excludes missing and “don’t know” responses to the survey question, “Is there a designated health/public health representative on the food policy council, food security coalition, or other community group working to increase access to healthy food?”

e Significant based on the 95% CI not including the null value of 1.

f Includes 425 municipalities with a yes response to the survey question, “Does your jurisdiction have a local or regional food policy council, food security coalition, or similar entity?” Excludes missing and “don’t know” responses to the survey question, “Is there a designated community representative on the food policy council, food security coalition, or other community group working to increase access to healthy food?”

## Discussion

Our study updates associations previously found in the 2014 CBS-HEAL survey showing the positive association between the presence of an FPC and the existence of health-promoting policies to support healthy food access (12). A unique contribution of our study is the potential importance of FPC membership composition; membership composition is important because of the role of FPCs in broad efforts to enact PSE changes that support healthy food access in communities.

The policies examined in our study align with the primary efforts reported by FPCs as of 2020 (10). Specifically, healthy food access, which includes healthy food financing and food and nutrition incentives at farmers markets, has consistently remained the most common policy priority among FPCs since 2014 (10). Antihunger and antipoverty work, which includes outreach and enrollment for the federal Supplemental Nutrition Assistance Program (SNAP) and other federal social assistance programs, increased to the second most common policy priority among FPCs, from 19% in 2014 to 53% in 2020 (10), aligning with our findings that municipalities with FPCs had significantly higher odds of having supports for EBT at farmers markets, farm stands, or green/produce carts. Food production, transportation, and distribution gained renewed interest in 2020, while the priorities of food procurement, land use planning, and food waste reduction and recovery decreased. As of 2020, 76% of FPCs engaged in at least 1 action to advocate for policy changes related to these priority issues (10).

Although there are reports that FPCs are a mechanism to achieve positive food system outcomes, limited empirical evidence supports this claim (6). This may be because FPCs view their work as “indirect, facilitative, and collaborative [making it] difficult to isolate the impacts of our specific efforts” (26), and determining cause and effect is difficult due to the complexity of food systems (20,27). Capacity for conducting evaluation studies is a challenge when almost one-third of FPCs report having no funding and an additional one-third report an annual budget of $10,000 or less (10). Furthermore, only 36% of FPCs reported having paid staff in 2020, which may limit the depth and breadth of their scope of work (10). This lack of resources may cause FPCs to focus more heavily on education and programs than on policy efforts (8,9,18,28). While education and programs likely contribute to the pathway to policy adoption, the outcomes from these efforts are difficult to systematically measure as a direct contributor to adopting policies. While our results showed a positive association between FPCs and existing policies, additional factors that our study did not measure may play independent or mediating roles in measuring the effectiveness of FPCs. One factor our study measured in relation to healthy food access policies is FPC membership composition.

Several studies have examined FPC membership composition (7,8,10,11,15,28). One study found that 63% of FPCs had members who were government or elected officials (9); our study found 44.0% and the previous study, in 2014, found 41% (12). Because some FPCs are embedded in government or receive in-kind support (eg, administrative help) from their local government, respondents may have inadvertently answered no to our survey question on local government membership; respondents may not view the FPC’s structural relationship to government as formal membership, which could account for the lower percentages reported. Additionally, respondents may not have known which category (government or public health) to use to categorize a government official working in public health (eg, director of health). The potential for different interpretations of the survey question could explain why our study did not find a significant association between reporting a government employee or elected official with policies to improve access to healthy foods. Previous research on FPC relationships with government and how this relationship influences the policy work of FPCs is mixed (15). Schiff (8) and Bassarab and colleagues (15) found that a strong tie to government can strengthen an FPC’s credibility and access to resources but can also undermine autonomy. The importance of government representation as members of FPCs depends on the community, available resources, and how food access priorities fit into the government agenda (3), factors that our survey did not measure.

Our study showed that having a health/public health representative was associated with having policies to improve healthy food access. Health/public health representatives can bring expertise, funding, coordination, transparency, and accountability (11,15). Types of contributions of health/public health representatives on FPCs range from serving as catalysts for setting agendas, offering resources such as meeting spaces or facilitation, and providing connections to other government resources (18). Furthermore, health/public health representatives are experts in their local government system and may have better access to data than other FPC members, enhancing understanding of food access issues (16) in their own municipalities. Dedicated health/public health members could bring the needed expertise and capacity to design and facilitate data collection and analysis for evaluation studies, particularly if these people are funded through their respective organizations with dedicated time to support their FPC membership.

The effectiveness of FPCs in achieving intended food systems outcomes can be enhanced by including diverse groups of community residents and other key food systems working partners (11,15,29–31). All community members in a food system have valuable expertise and experience that can contribute to the process of developing solutions to food-related problems (28). People who experience the effects of policy decisions can add to the effectiveness and equity of policy development and implementation and help increase participation and adherence to food-related programs while also addressing cultural needs (15,32). Our study showed that municipalities with greater than 20% of their population at or below the federal poverty level had significantly increased odds of having an FPC, which may indicate community-driven priorities and needs to address equity and structural food system issues.

Our data do not capture the extent to which community members are represented on FPCs versus the extent to which they actively participate in FPC decisions, which is an important distinction for building appropriate and lasting solutions (28,33). It is critical to balance the influence of experts and professionals with the community experience and knowledge of food systems issues, particularly since community members are traditionally excluded from the policy process (15). Ultimately, FPC members recognize that building a network of partnerships is central to the overall effectiveness of an FPC (28).

### Strengths and limitations

Our study has several strengths. A main strength is that the findings are nationally representative. A recent review of FPCs noted that almost all studies included in that review used a case study approach with primarily qualitative methods (19), which provide valuable contextual data but limit the generalizability of the results. Our study used quantitative methods to analyze national survey data, which may extend the generalizability of our findings and knowledge of FPCs. The application of quantitative methods to further evaluate single FPCs at a local level is lacking; such analysis is important in answering questions related to the effect of FPC activities (19). Thus, another strength of our study was the ability to assess potential effects of FPCs on PSE change (6,11,14,15,19,20).

Our study also has several limitations. First, survey data were self-reported by city manager planners and similar representatives, which may have resulted in misclassification since we could not verify the presence of reported FPCs or policies (12). Confirming adoption of the policies and reassessing the associations examined here could be an area of future research. Respondents’ awareness of FPCs may have changed from 2014 to 2021, or respondents were possibly unaware of FPC or policy existence in their municipalities. Second, although the survey’s findings may not be generalizable to very small municipalities (≤1,000 people), the survey pilot demonstrated that municipalities with less than 1,000 people were least likely to have the policies included in our study and accounted for only 3% of the US municipal population (34). Third, because our study design was cross-sectional, we could not determine a causal, temporal link between FPCs and the existence of healthy food supports (12). Furthermore, FPCs may be more likely to exist where healthy food policies already exist. Additionally, we did not account for potential geographic clustering of similar priorities and any influence of neighboring municipalities with FPCs, which may have contributed to the observed positive associations. Fourth, we could not assess policy implementation from the survey responses. Fifth, despite being administered from May through September 2021, the survey was not designed to capture the context of the COVID-19 pandemic or the potential effects of the pandemic on the existence of FPCs or policies to support improving access to healthy foods. Like other surveys (15), our survey provides only a crude assessment of membership composition (ie, only sector representation), not the total number of members nor number of members representing each sector, or if and how members are selected. Finally, the survey asks about “food policy council, food security coalition, or other community group working to increase access to healthy food,” which does not allow differentiation between broader and single-issue focused groups on associations with the policies included. Single-issue groups may be different, for example, because they dedicate their efforts, resources, and expertise to a single issue, resulting in potentially larger effects on a specific food policy.

### Conclusion

FPCs are useful for developing community-informed policy and systems strategies to address the complexity of food systems that members may not be able to tackle alone (15). Our study extends previous research showing the positive association between the presence of an FPC and the existence of health-promoting policies that support goals related to reducing chronic disease. Additionally, our study highlights the potential importance of FPC membership composition, including health/public health and community representatives, and the various roles of members in health-promoting policies. FPCs can promote health equity by recruiting socioeconomically and demographically diverse groups to join their councils and participate in policy development to help ensure acceptability (20). As FPCs continue to undertake work on food systems policy to maximize benefits for population health and for groups that experience health and economic disparities, health professionals, public health professionals, and community representatives have the expertise and skills to help FPCs evaluate and capture data on the effect of their collective work.
